# Characterization of oral microbiota in marmosets: Feasibility of using the marmoset as a human oral disease model

**DOI:** 10.1371/journal.pone.0207560

**Published:** 2019-02-07

**Authors:** Sachiko Takehara, Jorge L. Zeredo, Yasuhiro Kumei, Kensuke Kagiyama, Kazumasa Fukasawa, Akiko Oshiro, Masayuki Ueno, Noriko Kojimahara, Shunsuke Minakuchi, Yoko Kawaguchi

**Affiliations:** 1 Department of Public Health, Tokyo Women’s Medical University, Tokyo, Japan; 2 Department of Oral Health Promotion, Graduate School of Medical and Dental Sciences, Tokyo Medical and Dental University, Tokyo, Japan; 3 Graduate Program in Health Sciences and Technology, University of Brasilia, Brasilia, Brazil; 4 Department of Biochemistry, Graduate School of Medical and Dental Sciences, Tokyo Medical and Dental University, Tokyo, Japan; 5 Clea Japan Inc., Gifu, Japan; 6 Department of Health Sciences, Saitama Prefectural University, Saitama, Japan; 7 Gerodontology and Oral Rehabilitation, Graduate School of Medical and Dental Sciences, Tokyo Medical and Dental University, Tokyo, Japan; Estacion Experimental del Zaidin - CSIC, SPAIN

## Abstract

With rapid aging of the world’s population, the demand for research, for a better understanding of aging and aging-related disorders, is increasing. Ideally, such research should be conducted on human subjects. However, due to ethical considerations, animals such as rodents and monkeys are used as alternatives. Among these alternative models, non-human primates are preferred because of their similarities with humans. The small South American common marmoset (*Callithrix jacchus*) may offer several advantages over other non-human primates in terms of its smaller size, shorter life-span, and dental anatomy identical to humans. The purpose of this study was to determine the viability of using the marmoset as a human oral disease model. We collected saliva samples from eight marmosets and eight human subjects. Prokaryotic DNA was extracted from the saliva samples, and 16S bacterial rRNA gene sequencing was performed on each of the samples. Our results indicated that the types of oral microbiomes detected among human and marmoset samples were nearly indistinguishable. In contrast, the oral microbiomes of our human and marmoset subjects were distinctly different from those reported for rats and dogs, which are currently popular research animals. The oral microbiomes of marmosets showed greater diversity than those of humans. However, the oral microbiota of marmosets exhibited less variation than those of humans, which may be attributed to the fact that the marmoset subjects were kept in a controlled environment with identical lifestyles. The characteristics of its oral microbiota, combined with other technical advantages, suggest that the marmoset may provide the best animal model thus far for the study of oral health. This study characterized the oral microbes of the marmoset, thereby providing information to support future application of the marmoset as a model for age-related oral disease.

## Introduction

Aging is a complex phenomenon affecting all organs of the body. In the field of basic science, experimental subjects such as cells, worms, flies, and rodents have provided extensive groundwork for aging research [1]. However, taking this science from the bench to the bedside requires studies in more complex species with physiology and aging processes that resemble those of humans more closely. Therefore, there is an increasing demand for research using non-human primates, which have higher phenotypic similarities and share a high genetic homology with humans [2]. The small South American common marmoset (*Callithrix jacchus*) offers a number of advantages over other non-human primates given their relatively small size and shorter life-span [2]. Consequently, the marmoset has been used as a primate model of age-related diseases such as Parkinson’s disease, respiratory diseases, and infectious diseases.

Due to rapid aging of the world’s population, an increasing number of studies have articulated the importance of maintaining oral health. For instance, the prevalence of periodontitis increases with age, as shown by epidemiological studies [3, 4]. Indeed, ageing results in alterations to the immune system leading to dysregulation of immune responses [5]. Nevertheless, the increased susceptibility to periodontitis in older people cannot be attributed simply to the decline of the immune system, as the patterns of change caused by ageing are complex [6]. Ebersole et al. reported that antibodies levels are lower and microbial components are altered in older patients, concluding that ageing has a minimum effect on the adaptive immune response [7].

A substantial number of studies have examined the influence of oral health status, especially the existence of periodontitis, on general health. Examples include the association between the severity of periodontitis and cardiovascular disease, diabetes mellitus, and neurodegenerative diseases [3, 8–12]. Periodontitis and caries are major infectious diseases of the oral cavity and are closely related to the presence of specific oral bacteria. These oral microbes have been studied extensively with great interest. More than 700 indigenous bacterial species inhabit the human oral cavity [13] and they form microbial communities within dental plaques and tongue coatings. As with other non-human primates, the oral anatomy of marmosets share common characteristics with humans. For example, the adult marmoset has two incisors, a short-tusked canine tooth, three premolars, and two molars in each oral quadrant, with a total of 32 teeth [14]. Some studies have detailed the oral microbes of non-human primates [15, 16]. In fact, oral bacteria from the marmoset have been studied less extensively, although the marmoset is considered a powerful model for scientific research due to behavioral and cognitive capacities similar to those of humans [17–19]. The purpose of this study was to examine the possibility of using marmosets as a human oral disease model. The present investigation was designed to reveal the major bacteria in the oral cavity of marmosets and examine the similarity with those in the human oral cavity using targeted sequencing of the microbial 16S rRNA gene.

## Materials and methods

### Study subjects

Eight healthy common marmosets aged between 10 and 18 years were selected from the colony at Clea, Japan (Gifu, Japan). Human subjects were recruited among the patients who visited the Dental Hospital, Tokyo Medical and Dental University. Finally, eight healthy human subjects (male = 4, female = 4, age range: 37–61 years) were selected after excluding those with any prescribed medication, periodontitis, or systemic diseases.

### Saliva collection

Marmosets were fasted on the day of saliva collection to obtain undisturbed oral microbiota from each animal. Saliva samples were collected by probing sterilized cotton swabs (JCB Industry Limited, Tokyo) in the oral cavity and allowing the marmosets to habitually chew on the cotton swabs. After 2–3 minutes, the cotton swabs were removed and suspended in 1 ml of sterile distilled water in sterile 1.0-ml tubes. The tubes were then stored at −80°C, until assayed for DNA analysis.

As for human subjects, they were asked to refrain from eating, drinking, brushing their teeth, and rinsing their mouth on the day of saliva collection to obtain undisturbed oral microbiota from each subject. Saliva was obtained by requesting them to spit saliva into sterile tubes while sitting in an upright position in a chair; saliva samples were frozen immediately and then stored at −80°C until analysis. To minimize the effects of circadian rhythm, samples from the two species were collected between 9:00 a.m. and 11:00 a.m.

### Oral examination

All subjects underwent a standard oral examination. Oral examinations for marmosets and humans were conducted by registered dentists. Data such as types of teeth present, teeth condition (sound, decayed, or missing), and gingival inflammation were collected. During the oral examination, the marmosets were manually held by the veterinary staff using hands and arms. The mouth was kept open by placing a wooden chopstick between the marmoset’s posterior teeth. To minimize physical and emotional distress to the animals, anesthesia or special restraint devices were not used.

### DNA isolation and 16S rRNA gene sequencing analysis

DNA was extracted using a NucleoSpin Soil (Macherey-Nagel, Duren, Germany) according to the manufacturer’s instructions. The quantity and quality of extracted DNA were determined using a NanoDrop spectrophotometer (Thermo Fisher Scientific Inc., Tokyo, Japan), Quant-iT dsDNA HS Assay Kit (Thermo Fisher Scientific Inc., Tokyo, Japan), and agarose gel electrophoresis.

The fusion primers 341F (5’-TCGTCGGCAGCGTCAGATGTGTATAAGAGA CAGCCTACG GGNGGCWGCAG-3’) and 806R (5’-GTCTCGTGGGCTCGGAGATGTGTATAAGAGACAG GGACTACHVGGGTWTCTAAT-3’) with dual index were used for amplifying the V3–V4 regions of the bacterial 16S rRNA gene using a melting temperature of 50°C and 28 cycles. The sequencing was performed on the Illumina MiSeq platform (Illumina, San Diego, California, USA).

For clustering of sequences into operational taxonomic units (OTUs), CD-HIT-OTU (version 0.0.2) was utilized. Sequences were clustered into OTUs at 97% similarity using CD-HIT-OTU software. Taxonomic assignments for the 16S rRNA was performed using the RDP classifier program (version 2.2). A total of 51,135 sequences remained and were automatically used for clustering after quality control screening.

### Ethics statement

No animals were sacrificed during this study. All animals were cared for according to the protocol approved prior to the start of the study by the Animal Welfare Committee of Clea Japan Co. (Tokyo, Japan). All study animals were kept on a 12-hour light/dark cycle (7 a.m./7 p.m.) and were individually housed in isolated cages. The size of the cage was as follows: length 39 cm, width 55 cm, and height 70 cm. All were fed with commercially available monkey food for breeding and stud feed CMS-1M (FEED ONE CO., LTD., Japan) twice a day. Daily care was provided by the same staff, and the veterinary staff took care of the animals in case of any health problems. The veterinary staff also examined the conditions of animals before and after the study. As for the human subjects, the study was performed in accordance with the World Medical Association Declaration of Helsinki, and the study protocol and consent procedures were approved by the institutional ethics committee at Tokyo Medical and Dental University prior to commencement of the study (Approval No. D2015-606).

### Statistical analysis

Statistical analyses were performed using the Statistical Package for Social Sciences (SPSS) for Windows, version 11.5 (SPSS, Inc., Chicago, IL, USA). OTU numbers, Shannon diversity index, Chao 1 diversity index, and unweighted UniFrac were calculated using QIIME (v. 1.8.0). The Mann-Whitney U test was used to compare the microbial composition between the two species. A two-sided p value < 0.05 was used as a level of statistical significance.

## Results

### Demographic characteristics

Human participants were provided written and verbal explanation with respect to the purpose of this study. The eight subjects signed the consent forms and agreed to enroll in the study. As for the animal subjects, eight healthy marmosets were randomly selected. Distribution of age among the human subjects and marmosets is shown in Table 1.

**Table 1:** List of subjects and their characteristics.

species	ID	age	gender	teeth present	Number of OTUs
human					
	h-1	41	Male	28	105
	h-2	49	Male	27	132
	h-3	61	Male	25	91
	h-4	38	Male	28	124
	h-5	37	Female	24	75
	h-6	42	Female	28	104
	h-7	42	Female	24	98
	h-8	51	Female	20	84
					Median: 101, Max:132, Min: 75
marmoset					
	m-1	10	Male	19	118
	m-2	12	Male	16	117
	m-3	15	Male	13	116
	m-4	18	Male	20	115
	m-5	12	Female	13	139
	m-6	12	Female	23	122
	m-7	15	Female	23	123
	m-8	12	Female	12	124
					Median: 120, Max:139, Min: 115

### Oral condition

The oral conditions compared are shown in Table 1. The number of present teeth among marmosets and humans varied from 12–23 and 20–28, respectively. Marmosets had a significantly smaller number of present teeth and a larger number of missing teeth than humans (p = 0.001). The main reason marmosets lost their teeth was habitual behavior such as chewing on hard wood. No subjects, in either the human and marmoset groups, had decayed teeth. All marmosets had dental calculus deposited on more than one of their teeth, and gingival inflammation (swelling or bleeding) was observed at multiple sites. As for human subjects, none had any teeth with periodontal pockets.

### Saliva microbiome diversity

**Fig 1:**
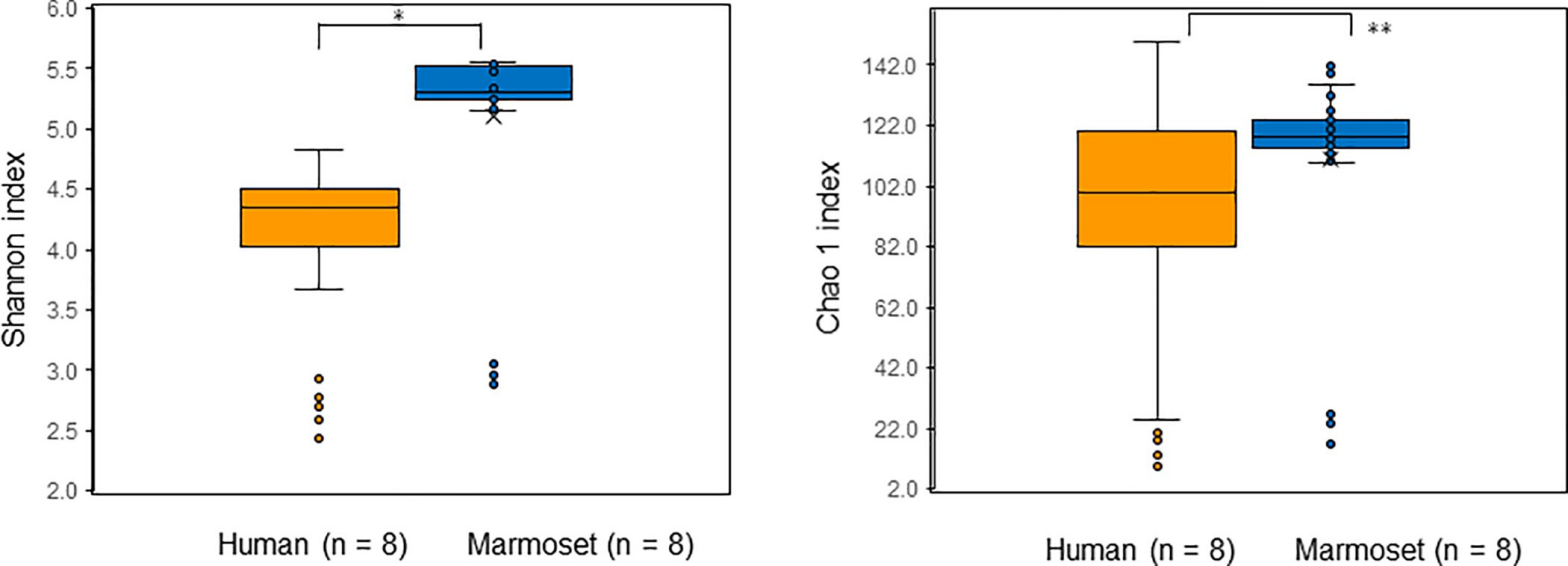
Diversity analysis for humans and marmosets based on the bacteria abundance distribution for each individual at OTU level. (A) Shannon diversity. Vertical axis indicates the diversity in number of distinct OTUs. (B) Chao 1 index diversity. Vertical axis indicates the richness estimates in number of distinct OTUs. *: p<0.0001, **: p = 0.0013.

The distribution of the number of OTUs are shown in Table 1. The number of OTUs did not show any significant differences between two species (p = 0.052), however, the median, minimum, and maximum values of the marmosets were higher than those of humans. To characterize the phylogenetic composition of bacterial communities in the oral cavity, the α-diversity of the microbiota were examined. The marmosets had a significantly higher Shannon’s index and Chao 1 index than humans (Fig 1A and 1B, p < 0.001, p = 0.0013, respectively), indicating a higher α-diversity of oral bacteria in marmosets.

**Fig 2:**
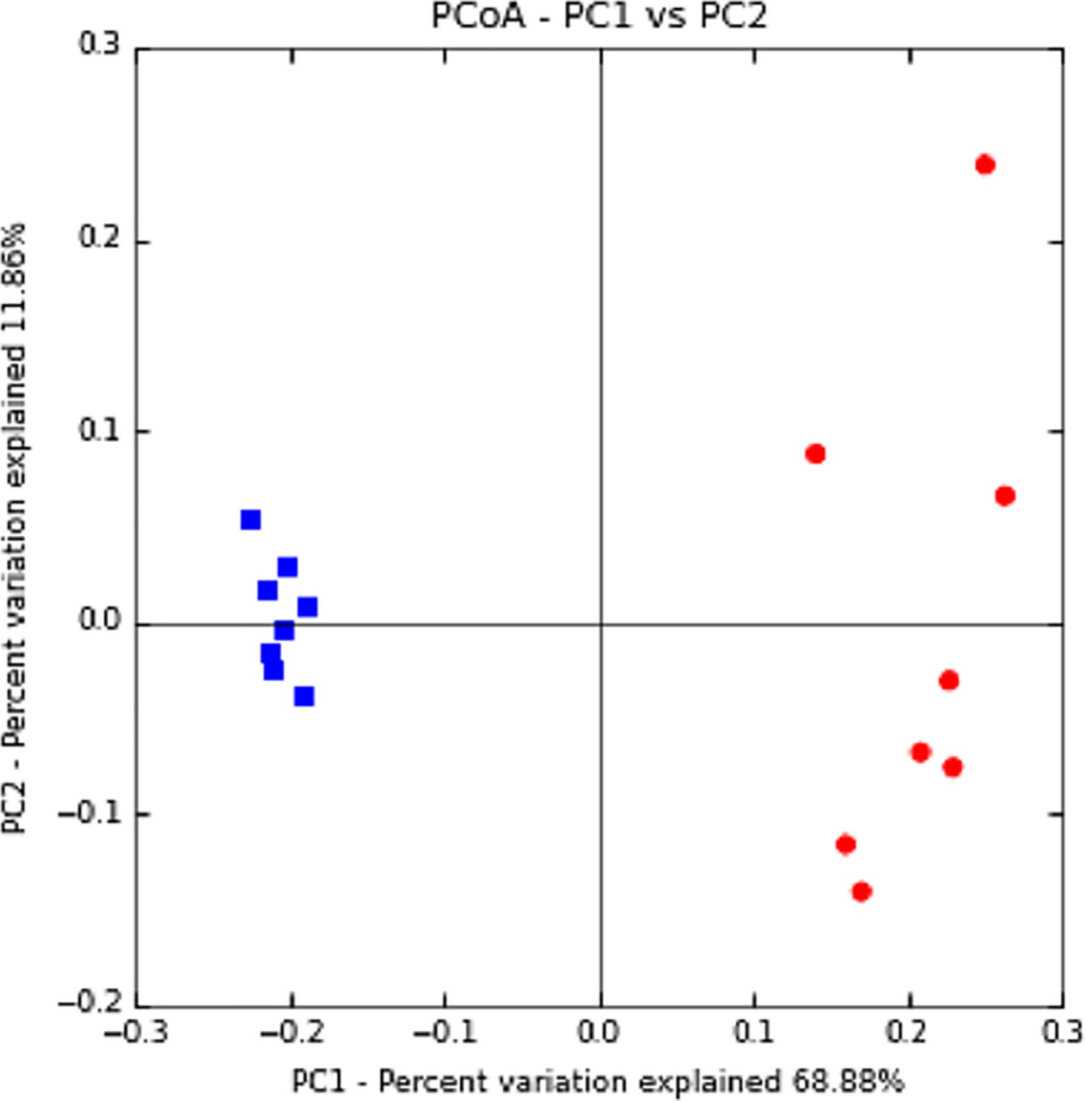
A principal-coordinate analysis plot of the oral microbiota based on the results of the unweighted UniFrac metric. Scatter plot of principal coordinate 1 (PC1) versus principal coordinate 2 (PC2) is shown. The first axis (PC1) explains the maximum amount of variation present, followed by the second axis. Percentages shown are percentages of variation explained by the components. Each point represents a single sample, and the distance between points represents the compositional difference of the samples from each other. ■: marmoset, ●: human.

The overall bacterial community composition was compared using the UniFrac. UniFrac represents a phylogeny-based distance metric ranging from 0 (identical bacterial communities) to 1 (completely different). A principal-coordinate analysis plot based on unweighted UniFrac values revealed distinct clustering of samples from marmosets and humans, indicating the difference in microbial compositions between the two species (Fig 2). As for their spatial distributions, samples from marmosets were densely clustered, whereas those from humans were loosely clustered. This tendency indicates that marmosets had smaller individual variations of their oral microbes.

**Fig 3:**
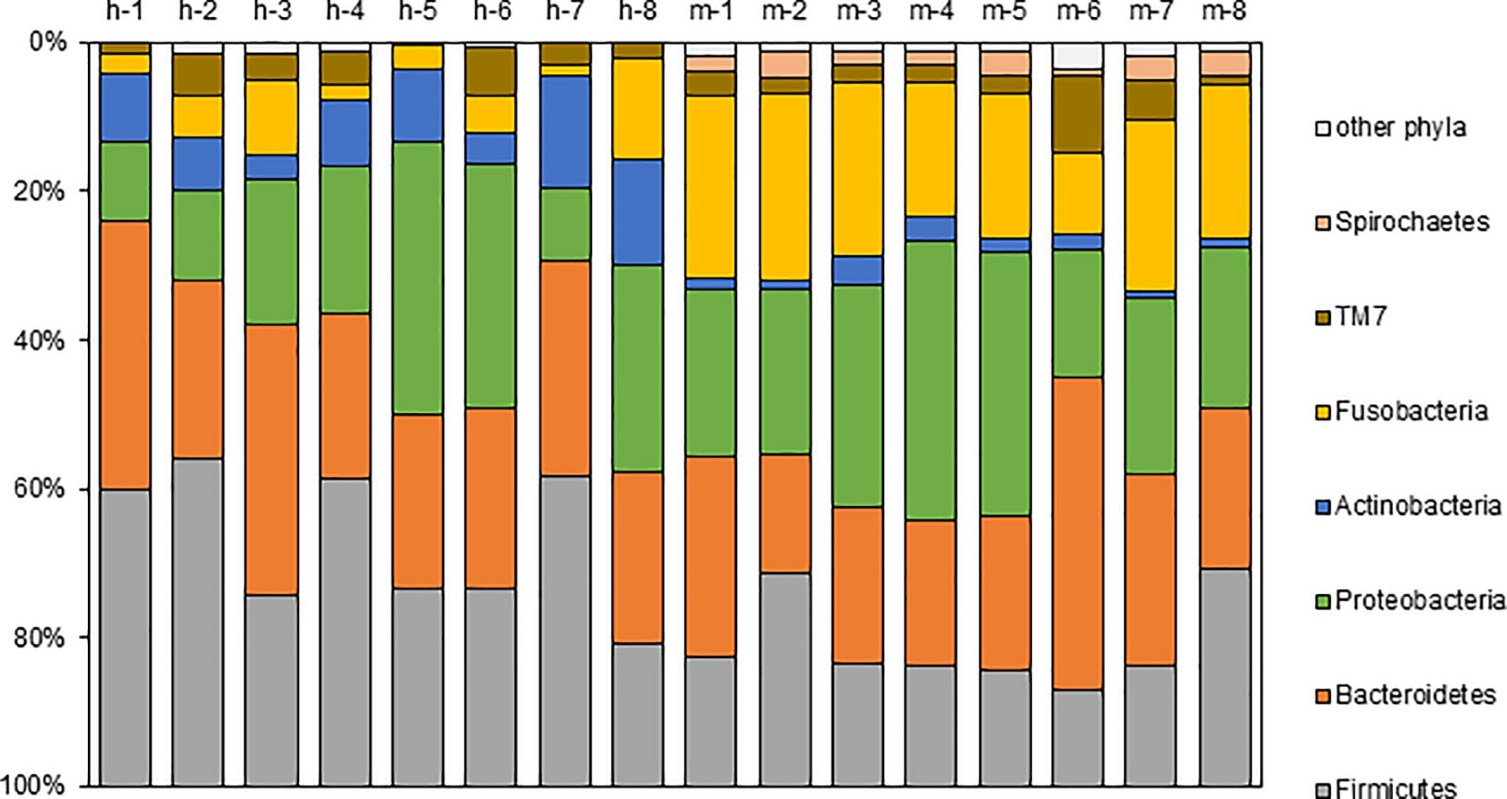
Microbial composition at the phylum level. Samples are represented along the horizontal axis, and relative abundance is denoted by the vertical axis. h-1 –h-8: human, m-1 –m-8: marmoset.

**Fig 4:**
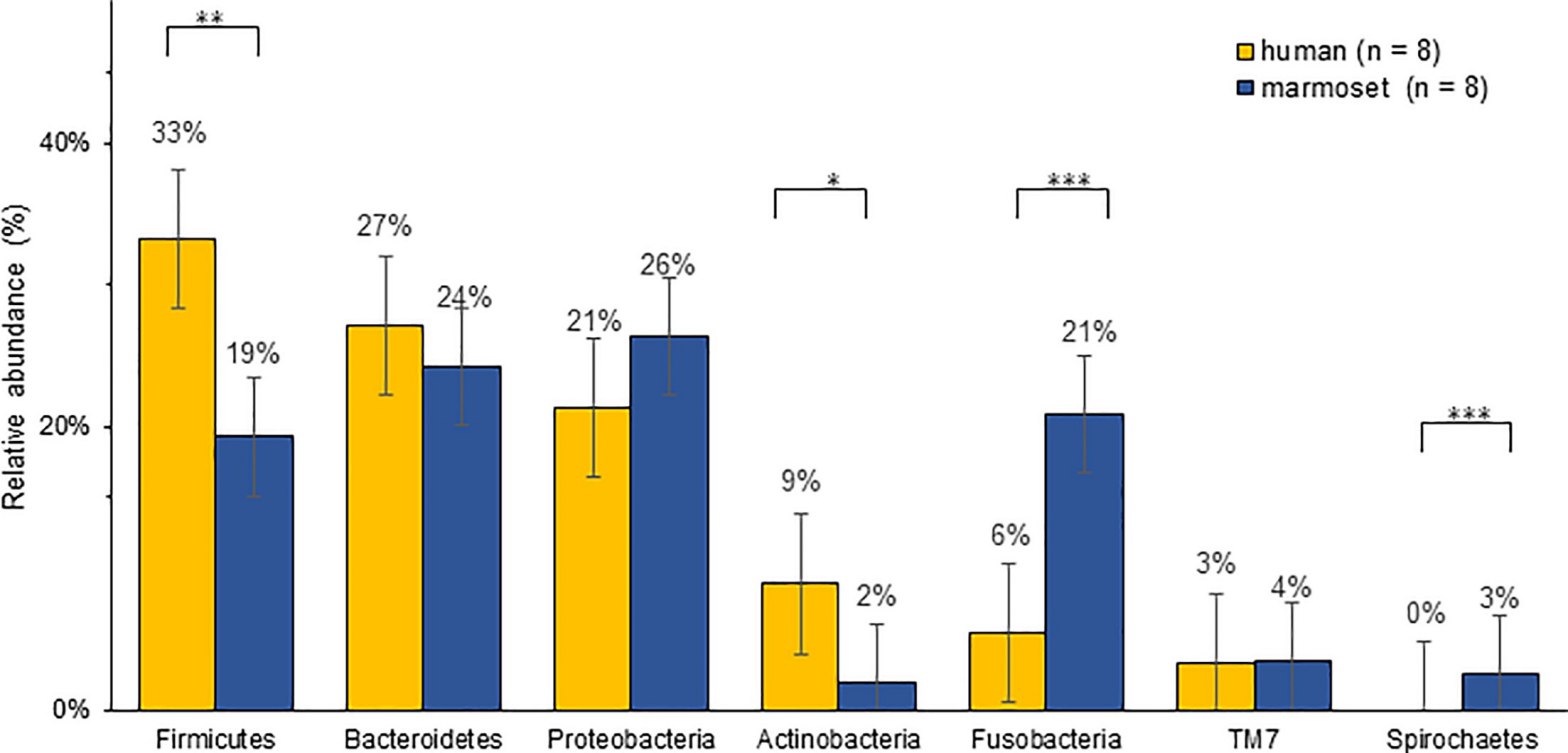
Relative abundance of bacteria (phylum level). *: p = 0.002, **: p = 0.004, ***: p < 0.0001.

Furthermore, taxonomic distribution of the numerically abundant bacteria derived from the 16S rRNA gene sequences in all samples were examined (Fig 3). Most of the sequences obtained in this study were assigned to seven bacterial phyla—*Firmicutes*, *Bacteroides*, *Proteobacteria*, *Actinobacteria*, *Fusobacteria*, *TM7*, and *Spirochaetes*. The other phyla, *Tenericues*, *Synergistetes*, and SR1 were detected in traceable small percentages among the whole oral microbiome in our study samples. The average relative abundance of these seven phyla is shown in Fig 4. *Firmicutes*, *Bacteroides*, and *Proteobacteria* were the major phylotype in human saliva, which accounted for 33%, 27%, and 21% of relative abundance on an average, respectively. The fourth abundant phylotype was *Actinobacteria*, which accounted for 9%. On the other hand, in the marmoset samples, *Proteobacteria*, *Bacteroides*, *Fusobacteria*, and *Firmicutes* were the major phylotype, which accounted for 26%, 24%, 21%, and 19%, respectively. Their relative abundance was statistically examined using the Mann-Whitney U test. Compared with humans, *Actinobacteria* and *Firmicutes* were statistically less abundant in marmosets (p = 0.002, p = 0.004), and *Fusobacteria* and *Spirochaetes* were statistically more abundant in the marmosets (p < 0.001).

## Discussion

In this present study, we analyzed oral microbes from marmosets and humans for comparison. This is the first known report revealing the viability of marmosets as an animal model for oral diseases and oral physiology. Our study has demonstrated important similarities between oral microbes in marmosets and humans, particularly in relation to microbiome phyla, and revealed that oral microbes in marmosets exhibit higher diversity with less individual variations than human oral microbes.

Anatomically, both humans and marmosets have common dental physiology in terms of the number and types of teeth [14]. A marmoset’s life-span is known to be between 16 and 21 years [18, 20, 21]—approximately one-seventh of a human life-span; which can be useful for interventional or observational studies investigating physiological and functional changes accompanied with aging. This versatility also can provide an opportunity for further studying the association between the quality of life and poor oral health, such as the increased incidence of aspiration pneumonia, cognitive impairment, and frailty, of elderly individuals [22]. Aging is a multi-factorial phenomenon and no two individuals experience the same age-related decline. It is this relationship between the aging process and oral health that can be examined in the short life-span of the marmoset, allowing us to observe the effect of poor oral health on the quality of life in a much more manageable time span.

This study revealed that oral microbe samples from marmosets showed relatively higher diversity at the OTU level since the difference is close to the statistical level. Although, in general, the oral microbiome varies dynamically over one’s life-time, this diversity is known to be maintained via healthy oral conditions through an optimal balance between the oral microbiota and the host’s immune system [23]. Any onset of periodontitis, dental caries, or even smoking may influence bacterial diversity [24]. In addition, this diversity decreases when mammal subjects experience chronic illness such as liver or gastrointestinal diseases [25, 26]. Both groups of our study subjects were healthy without any prescribed medication, diagnosed chronic diseases, or smoking habit. For marmosets, we selected those that were 10 years of age or older. With respect to their reported life-span [18], our marmoset subjects were categorized as being in old-age. Age, being one of the main risk factors for general prevalent diseases [27], renders susceptibility to infections by a compromised immune system. If this age factor reflects the host’s health condition [28], our senior-aged marmoset samples should have lost oral microbiome diversity. Quite contrary to this, we observed that they maintained a high microbial diversity.

We found important similarities in the salivary microbiome between humans and marmosets. Five of the most abundant phyla in humans, *Firmicutes*, *Bacteroidetes*, *Proteobacteria*, *Actinobacteria*, *and Fusobacterium*, were detected in both humans and marmosets. This result is consistent with those reported in previous human and non-human-primate studies [16, 29], which further supports the notion that the marmoset may indeed be used in translational studies to model human health and disease. On the other hand, *Spirochaetes–*reported to include species of the genus *Treponema*,—were detected in all samples of the marmoset subjects, but not in the human subjects. This may be attributed to the fact that the marmosets in this study were old, with calculus deposited on their teeth, implicating the existence of periodontitis or gingivitis, whereas the humans were healthy middle-aged subjects without any significant oral or systemic disease. In previous studies, *Spirochaetes* were reported to be detected in the saliva of patients with oral cancer, periodontitis, and systemic diseases [25, 26, 30].

Marmoset subjects showed inflammation of their gum, but human subjects did not. This explains why the phylum *Spirochaetes–*which is generally found in root canal infections, gingivitis, and periodontitis [21]–was detected in our marmosets’ samples, but not in the human samples. Previous studies reported clear differences among populations living under different geographical conditions [31, 32]. The differences were reportedly attributed mainly to differences in the lifestyles, dietary habits, as well as sanitary and socioeconomic factors [33, 34]. With respect to individual variation, oral microbes of marmosets had less variety in their oral microbiota compared to the human data, which indicated higher variation. This difference in variation can be attributed to the fact that the marmoset subjects were kept in a controlled environment with identical lifestyles (i.e. marmosets were kept in similar cages and fed with identical food).

Laboratory rats, such as Wistar rats, and dogs have been widely used in scientific experiments as models for human oral disease. The oral microbiomes of laboratory rats [35] and dogs [36] have been reported. Oral microbiomes of canines are similar to those of humans, nevertheless, a significant gap with respect to phylum differences exists. As for rats, distribution of the oral microbiota is reported to be even more different than that of humans. In contrast, the marmoset oral microbiome is much closer to that of humans, as compared to the reported microbiota of the rat and dog.

A major limitation of this study was that our sample consisted exclusively of old-aged marmosets. Although we selected marmosets with a wide age range (10 to 18 years), our sample turned out to be surprisingly homogeneous in terms of the oral microbiome and oral-health status. Therefore, we could not determine any associations between these factors (age, microbiome, and health/disease) within our sample. Nevertheless, it is reasonable to assume that such associations not only exist, but also that they can be teased out in future studies by including marmosets of younger age-groups and by collecting more detailed data on the marmosets’ oral health status, such as the severity of gingival inflammation, depth of periodontal pockets, and extent of dental decay. Such studies shall reinforce the feasibility of the marmoset as an ideal, promising oral health model for future research.

In the context of the marmoset’s dental physiology, shorter life-span, and smaller body mass, our findings suggest that the marmoset could be a model for studying general oral health and its relationship with aging. In conclusion, this study is the first to describe and discuss the oral microbes of the marmoset, thereby providing important information for the application of the marmoset as a model in studies of age-related oral diseases.
